# Serum biomarkers of collagen turnover as potential diagnostic tools in diffuse systemic sclerosis: A cross-sectional study

**DOI:** 10.1371/journal.pone.0207324

**Published:** 2018-12-03

**Authors:** Pernille Juhl, Anne-Christine Bay-Jensen, Morten Karsdal, Anne Sofie Siebuhr, Nathalie Franchimont, Juan Chavez

**Affiliations:** 1 Biomarkers and Research, Nordic Bioscience, Herlev, Denmark; 2 Department of Biomedical Sciences, Copenhagen University, Copenhagen, Denmark; 3 Clinical Development, Biogen, Cambridge, Massachusetts, United States of America; Keio University, JAPAN

## Abstract

**Background:**

Systemic sclerosis (SSc) is characterized by excessive fibrosis throughout the body. This leads to the release of extracellular matrix (ECM) fragments into circulation, where they may be quantified as biomarkers. The objectives were to investigate levels of ECM turnover biomarkers and the diagnostic power of these.

**Methods:**

Diffuse SSc patients (n = 40) fulfilling the ACR/EULAR 2013 classification criteria and asymptomatic controls were included. Patients were divided into early (<2 years of symptoms; n = 20) and late (>10 years of symptoms; n = 20) diffuse SSc. Biomarkers of type I (C1M), III (C3A, C3M), IV (C4M), V (C5M) and VI (C6M) collagen degradation and type I (PRO-C1), II (PRO-C2), III (PRO-C3), IV (PRO-C4), V (PRO-C5) and VI (PRO-C6) collagen formation were measured in serum.

Repeated measures ANOVA was used to test for differences in biomarker levels and the area under the receiver operating characteristic curve (AUC) was used to investigate the ability of the biomarkers to separate groups.

**Results:**

In early diffuse SSc, formation biomarkers of type III, IV, V and VI collagen were significantly increased compared to asymptomatic controls (p<0.0001). Moreover, in early diffuse SSc formation biomarkers of type III, V and VI collagen were significantly increased compared to late diffuse SSc (p = 0.0006, 0.003 and 0.004, respectively). Type I (p<0.0001), III (C3M: p = 0.001, and C3A: p = 0.02), IV (p<0.0001) and VI (p<0.0001) collagen degradation biomarkers significantly increased in early diffuse SSc compared to controls. C4M, C6M, PRO-C4, PRO-C5 and PRO-C6 had an AUC of >0.85 when assessing asymptomatic controls vs. diffuse SSc. Biomarkers of type VI collagen (PRO-C6 and C6M) turnover had the best separation with an AUC’s of >0.90.

**Conclusion:**

Formation biomarkers of ECM turnover were shown to be significantly different between asymptomatic controls and diffuse SSc. This pilot study suggest that serological biomarkers of the ECM turnover is potentially applicable in SSc.

## Introduction

Systemic sclerosis (SSc) is a rare, multisystem autoimmune disease of unknown etiology. One of its main characteristics is fibrosis of the skin and internal organs. The distribution of skin involvement is used to stratify patients into limited and diffuse cutaneous SSc, where diffuse cutaneous SSc is characterized by skin involvement of the trunk, face, proximal, and distal extremities[1]. Diffuse SSc is a more aggressive disease compared to limited cutaneous SSc with increased organ involvement and mortality[2–4]. SSc is thought to progress in two phases; an early, very active period followed by a slower progressing period.

Mammalian skin consists of several distinct layers. The outer most layer is the epidermis and the basal layer of the epidermis is attached to a basement membrane that overlays the connective tissue layer, known as the dermis. The predominant extracellular matrix (ECM) component of the skin is collagen. The two major collagens found are type I and III collagen, which are mainly found in the dermis[5]. Type IV, V and VI collagen are found at minor concentrations in the skin. Type IV collagen is located in the basement membrane in the junction of the epidermis and dermis[6]. Type V collagen is co-located with type I and III collagen in the dermis, where it plays an important role in regulating the fibrils[7]. Type VI collagen is likewise distributed throughout the dermis[8]. Dermal accumulation of type I and III collagen and up-regulation of other ECM proteins, including fibronectin, osteopontin, and SPARC, are observed during dermal fibrosis[9].

ECM remodeling releases a vast number of protein fragments into circulation. These fragments include both pro-peptides and protease generated degradation fragments of the ECM proteins. They describe the status of the ECM from where they originate and can be used to assess ECM turnover, i.e. natural tissue remodeling and diseases as fibrosis. The shifted balance in tissue remodeling during different diseases can be assessed by these protein fragments.

Several collagen fragments have been identified as possible diagnostic, prognostic, and predictive biomarkers in various arthritic and fibrotic diseases[10–17]. The pro-peptide of type III collagen (PRO-C3) has been shown to be diagnostic of liver fibrosis[10], while the degradation of type III collagen (C3M) has diagnostic capacities for idiopathic pulmonary fibrosis[11], rheumatoid arthritis (RA)[12] and ulcerative colitis[13]. The pro-peptide of type IV collagen (PRO-C4) has shown to be diagnostic of liver fibrosis[14], while the matrix metalloproteinase (MMP) degraded type IV collagen (C4M) has shown to be diagnostic of idiopathic pulmonary fibrosis (IPF)[15]. The pro-peptide of type V collagen (C5M) has shown diagnostic relevance with liver fibrosis[16] and to be a possible diagnostic tool for ankylosing spondylitis[17]. A type VI collagen degradation biomarker (C6M) have shown to have prognostic capacity in IPF[11]. MMP-degraded type I collagen (C1M) has shown to be predictive of radiographic progression in RA[18].

SSc is a multi-organ disease, known to affect joints, the gastrointestinal system, lungs, and liver. Biomarkers of these organs may be useful in SSc as diagnostic or predictive biomarkers. Such biomarkers have been briefly explored in SSc, where differences in biomarker levels between healthy controls and SSc has been observed for different collagen formation biomarkers[19–21]. PINP and PIIINP have been examined in clinical studies of SSc with varying results. Studies have shown that PINP and PIIINP do not consistently correlate with changes in modified Rodnan skin score (mRSS)[20, 22, 23].

There is an unmet need of objective serological biomarkers that can stratify patients. A better understanding of disease progression will aid in the development of better treatments. Investigations so far have only scratched the surface, but indications that serological biomarkers may be of value have been presented.

The objective of this study was to investigate the level of collagen protein fingerprint biomarkers in diffuse SSc (early and late) compared to asymptomatic controls and determine the diagnostic value of these biomarkers. This was done by quantifying the ECM biomarkers in a cross-sectional study comprising early and late diffuse SSc patient and asymptomatic controls.

## Methods

### Patients

Forty SSc patients were identified from the University of Pittsburgh Medical Center and University of Pittsburgh Scleroderma center observational cohort study, which consists of prospectively collected data and serum samples. Serum samples were collected from study subjects and stored at -80°C until measured. All SSc patients met the 2013 combined ACR/EULAR criteria[24], were > 18 years at the time of first visit, and had diffuse disease, defined as skin thickening proximal to the elbows and knees. Of the diffuse SSc patients, half (n = 20) of the patients had early stage disease defined as < 2 years since the first SSc symptom. Twenty patients were late stage SSc, with 10 or more years of symptoms, and no change in mRSS for at least six months prior to the serum sample. If diffuse SSc patients had interstitial lung disease, this was stable by forced vital capacity (FVC) for at least one year prior to the studied serum sample. Asymptomatic control sera were also taken from the Pittsburgh serum bank and did not have a personal or first-degree relative medical history of autoimmune disease.

All participants gave written, informed consent before inclusion in the study. The study was approved by the University of Pittsburgh institutional review board (approval number IRB0409097) and carried out in accordance with the principles of the declaration of Helsinki.

### Serological protein fingerprint biomarkers for ECM turnover

ECM degradation was assessed by type I collagen (C1M)[25], type III collagen (C3A;(unpublished) and C3M)[26], type IV collagen (C4M)[15], type V collagen (C5M)[17], and type IV collagen (C6M). ECM formation was assessed by the N-terminal type I collagen pro-peptide (PRO-C1)[27], the N-terminal pro-peptide of type II collagen (PRO-C2)[28], N-terminal pro-peptide of type III collagen (PRO-C3)[10], type IV collagen pro-peptide (PRO-C4)[29], C-terminal pro-peptide of type V collagen (PRO-C5)[16] and C-terminal pro-peptide of type VI collagen (PRO-C6)[30]. During assay development, cross-reactivity of the antibodies was examined. This was done by blasting the sequence of the antigen to ensure it is unique and the antibodies reaction to elongated and truncated peptides and no reactivity was found[30].

The described degradation and formation protein fingerprint biomarkers were assessed by validated competitive ELISAs. In summary, plates with streptavidin bound to the bottom, was coated with biotinylated peptide, corresponding to the specific epitopes to be assessed in the individual assay. This was incubated for 30 min at 20°C with agitation, followed by washing in washing buffer (20mM TRIS, 50mM NaCl, pH 7.2) five times. Twenty uL sample was added and 100uL antibody specific for the different epitopes in the individual assay was added. This was incubated either for 1 hour at 20°C, or 3 hours or 20 hours at 4°C. The plate was washed five times in washing buffer, before 100uL TMB was added to develop the ELISA. The enzyme reaction was stopped after 15 min by 0.1M H_2_SO_4_, before reading the plate at 450 nm with 650nm as reference. For PRO-C2 there was an additional step before TMB was added as 100 uL secondary antibody was added for 1 hour at 20°C. An overview of the assays used can be found in Table 1.

**Table 1:** Technical performance of the protein fingerprint biomarkers.

	Measuring range (ng/mL)	IC50 (ng/mL)	Inter-assay (%)	Intra-assay (%)
**C1M**	10–200	21–33	6.7–14	2.8–8.8
**C3A**	1.5–200	8–11	5.5–20	3–12
**C3M**	1–22	3–6	6.6–15.1	2–4.1
**C4M**	2.2–72	5–7	5–15	4–10
**C5M**	1–14	4.5–5.5	4.6–14	1–4.1
**C6M**	3–133.5	25–35	4–18	2–8
**PRO-C1**	7–258	45–60	3–13	1–8
**PRO-C2**	1.9–38.2	8.25	10	7
**PRO-C3**	1.3–58	5.5–8.5	8–12	1.8–9.3
**PRO-C4**	6–348.5	37.4–67.3	5.2–17.6	2.8–5.8
**PRO-C5**	32.5–1189	80–112	5–15	2–9
**PRO-C6**	0.4–67	3.5–5	3.4–12.4	1.1–5.3

### Statistics

Summary statistics were used to generate Table 2. Repeated measures ANOVA was used to test for differences in biomarker level between groups; asymptomatic controls, early diffuse, and late diffuse. Although biomarker data were not normally distributed, the assumptions of repeated measures ANOVA were fulfilled as the data were continuous and independent, there were no significant outliers, the distribution of the differences in the variables was approximately equal and the data showed sphericity. The area under the receiver operating characteristic curve (AUC) was used to investigate the ability of the biomarkers to identify cases from controls[31]; SSc groups from controls and early from late SSc groups.

**Table 2:** Clinical and demographic features of systemic sclerosis subjects and asymptomatic controls.

Characteristics	Early diffuse SSc (n = 20)	Late diffuse SSc (n = 20)	Asymptomatic controls (n = 20)	p-value
**Demographics**				
**Mean age (±SD) in years at the time sample**	54.0 ± 11.9	57.3 ± 14.7	45.5 ± 14.8	0.03
**Gender (% female)**	65%	85%	75%	0.34
**Disease and Exam Characteristics**				
**Median (IQR) disease duration in years (from first SSc-attributable symptom)**	0.8 (0.5, 1.2)	12.5 (10.6, 16.5)		<0.001
**Median modified Rodnan skin score (IQR)**	26.0 (10.5, 32.0)	7.0 (1.5, 9.5)		<0.001
**Internal organ system involvement**				
**Raynaud phenomenon**	90%	100%		0.49
**Pulmonary**	55%	90%		0.01
**Renal crisis**	5%	0%		0.31
**Cardiac**	0%	5%		0.31
**Gastrointestinal**	45%	75%		0.05
**Pulmonary hypertension**	0%	5%		0.31

SD: Standard deviation, IQR: interquartile range. For demographics, p-values were calculated by One-way ANOVA and for Disease and exam characteristics as well as internal organ involvement, p-values were calculated by t-test.

All data are shown as mean + standard error of mean (SEM), if not otherwise stated. All data analysis and graphical illustration was performed using GraphPad Prism version 6.00 for Windows, GraphPad Software, La Jolla California USA, www.graphpad.com or SAS 9.3 (SAS Institute, Cary, NC).

## Results

### Baseline demographics

The mean age of patients in the three groups were significantly different, with a younger asymptomatic control group (mean age: 45.5 years, p = 0.03). Early and late diffuse patients significantly differed on disease duration (p<0.001) and mRSS (p<0.001). Furthermore, a significant difference in pulmonary (p = 0.01) and gastrointestinal involvement was observed (p = 0.05; Table 2).

### Biomarker levels in the study population

**Fig 1:**
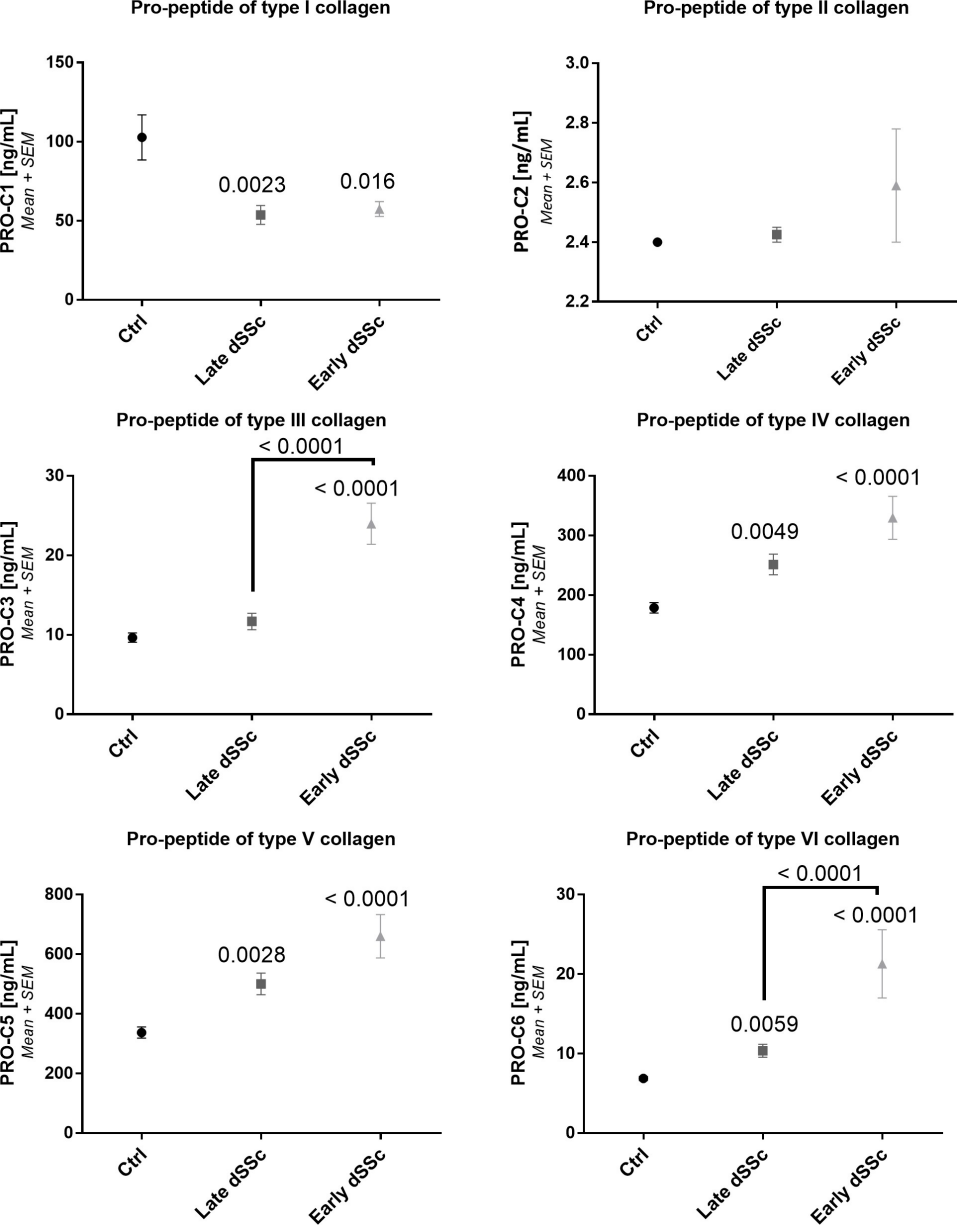
Collagen formation biomarker levels in asymptomatic controls and SSc groups. Type I collagen formation. B: Type II collagen formation. C: Type III collagen formation. D: Type IV collagen formation. E: Type V collagen formation. F: Type VI collagen formation. Repeated measures ANOVA (Kruskal-Wallis with Dunn’s multiple comparisons test) was used for statistical analysis, between asymptomatic controls, late and early diffuse SSc. Asymptomatic controls: n = 20, early SSc: n = 20 and late SSc: n = 20.

**Fig 2:**
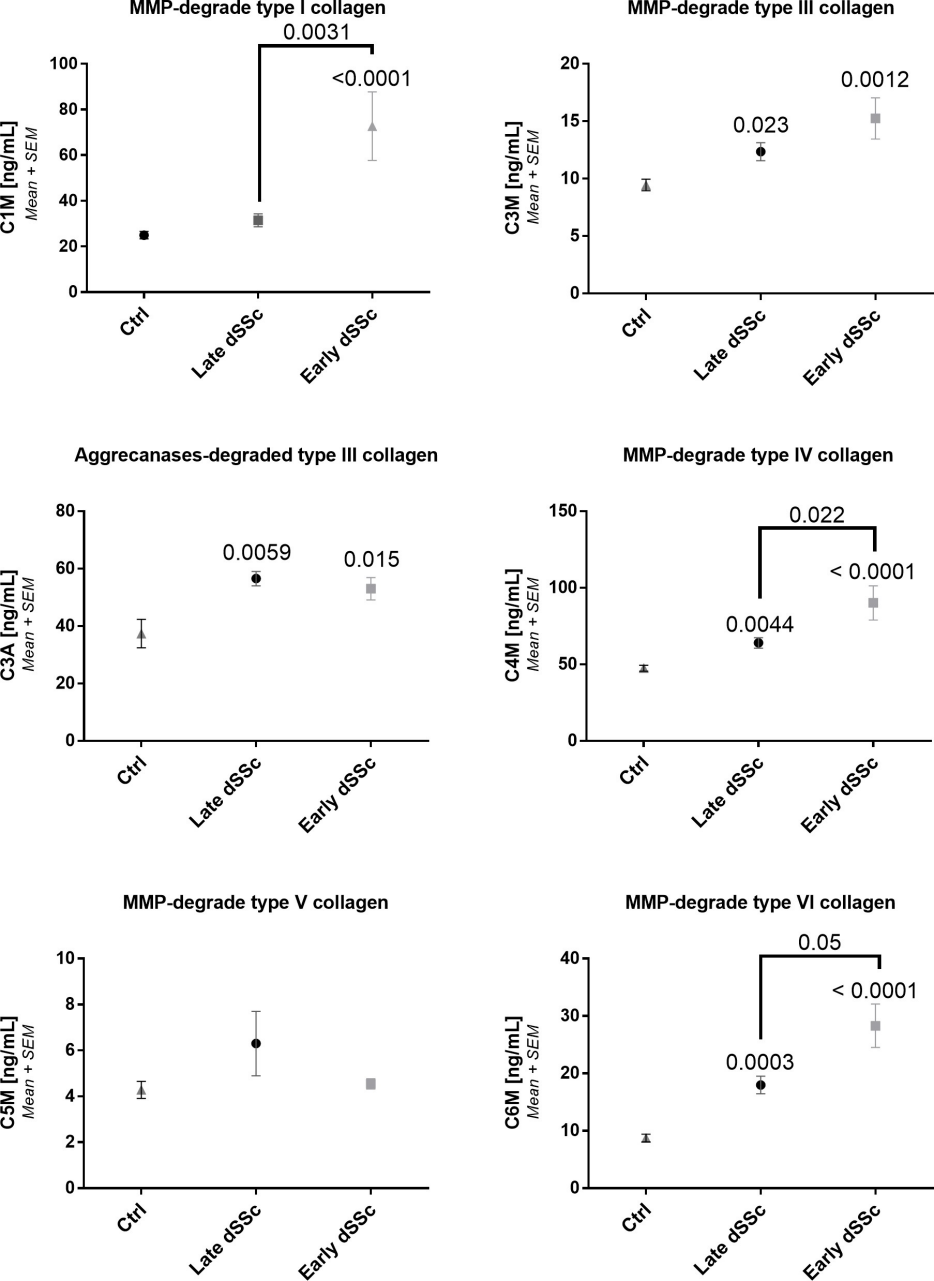
Collagen degradation biomarker levels in asymptomatic controls and SSc groups. A: Type I collagen degradation. B: Type III collagen degraded by aggrecanses. C: Type III collagen degraded by MMPs. D: Type IV collagen degradation. E: Type V collagen degradation. F: Type VI collagen degradation. Repeated measures ANOVA (Kruskal-Wallis with Dunn’s multiple comparisons test) was used for statistical analysis, between asymptomatic controls, late and early diffuse SSc. Asymptomatic controls: n = 20, early SSc: n = 20 and late SSc: n = 20.

One of the hallmarks of SSc is excessive ECM deposition in both the skin and internal organs. ECM turnover biomarkers can serologically assess tissue turnover. In both early and late diffuse SSc patients formation and degradation biomarkers were generally increased compared to asymptomatic controls (Figs 1 and 2). PRO-C3, PRO-C4, PRO-C5 and PRO-C6 levels (all p<0.0001) were found to be significantly increased in early diffuse SSc compared to asymptomatic controls (Fig 1). In early diffuse SSc a significant higher level of PRO-C3 (p<0.0001) and PRO-C6 (p<0.0001) was found compared to late diffuse SSc. Furthermore, PRO-C4 (p = 0.006), PRO-C5 (p = 0.004) and PRO-C6 (p = 0.01) was found to be significantly increased in late diffuse SSc compared to healthy controls. Contrary, PRO-C1 was significantly lower in early (p = 0.02) and late (p = 0.002) diffuse SSc compared to asymptomatic controls. I an earlier study[19], PRO-C3, PRO-C4, and PRO-C5 showed a significantly difference in the fold change of serum biomarker levels between SSc patients and asymptomatic controls, similar to this study.

In early diffuse SSc, C1M, C4M and C6M (p<0.0001), C3A (p = 0.015) and C3M (p = 0.001) were found to be significantly increased compared to asymptomatic controls (Fig 2). Furthermore, in late diffuse SSc patients a significant increase was found compared to asymptomatic controls in C3A (p = 0.0055), C3M (p = 0.025), C4M (p = 0.0057), and C6M (p = 0.0005). C1M (p = 0.02), C4M (p = 0.22), and C6M (p = 0.05) was found to be significantly increased in early diffuse SSc compared to late diffuse SSc. These data confirm the observed significant difference in C4M and C6M levels between SSc and asymptomatic controls as observed previously[19].

### Diagnostic evaluation

Due to the highly significant difference between asymptomatic controls and SSc in many of the biomarkers, the diagnostic capacity of the biomarkers was assessed (Table 3). The individual biomarkers of C4M, C6M, PRO-C4, PRO-C5, and PRO-C6 each demonstrated an AUC >0.85 to distinguish SSc vs asymptomatic controls. Similarly, C1M, C4M, C6M, PRO-C3, PRO-C4, PRO-C5, and PRO-C6 were calculated to have an AUC of >0.85 to classify asymptomatic controls vs. early diffuse SSc and C3A, C6M, and PRO-C6 had an AUC of >0.85 to distinguish late diffuse SSc vs. asymptomatic controls. PRO-C3, and PRO-C6 showed an AUC of >0.85, when comparing early diffuse vs. late diffuse. PRO-C6 had the highest diagnostic power with an AUC of 0.99 (specificity/sensitivity: 95/95) and C6M had an AUC of 0.94 (90/90) for asymptomatic controls vs. early diffuse SSc. When assessing the separation of asymptomatic controls vs. late diffuse SSc, an AUC of 0.87 (80/75) were observed for PRO-C6 and an AUC of 0.91 (95/75) for C6M.

**Table 3:** Area under the curve, with corresponding sensitivity and specificity of the biomarkers as diagnostic tools.

		Ctrl vs. Early SSc	Ctrl vs. late SSc	Early SSc vs. late SSc
**C1M**	AUC	0.87***	0.67	0.77
	Sensitivity/specificity %	85/75	80/45	70/65
	Likelihood ratio	3.4	2.3	2.2
**C3A**	AUC	0.83	0.86*	0.54
	Sensitivity/specificity %	89/80	89/84	45/68
	Likelihood ratio	4.4	5.6	1.4
**C3M**	AUC	0.82*	0.76	0.59
	Sensitivity/specificity %	75/75	75/70	65/55
	Likelihood ratio	3.0	2.5	1.4
**C4M**	AUC	0.93***	0.84**	0.71
	Sensitivity/specificity %	74/95	58/95	60/80
	Likelihood ratio	14.74	11.6	3
**C5M**	AUC	0.54	0.66	0.60
	Sensitivity/specificity %	50/69	50/74	88/47
	Likelihood ratio	1.6	1.9	1.7
	AUC	0.94***	0.91***	0.68
**C6M**	Sensitivity/specificity %	90/90	75/95	65/70
	Likelihood ratio	9	15.0	2.2
**PRO-C1**	AUC	0.77***	0.80	0.57***
	Sensitivity/specificity %	70/75	70/70	65/55
	Likelihood ratio	2.8	2.3	1.4
**PRO-C3**	AUC	0.91***	0.61	0.86***
	Sensitivity/specificity %	85/90	75/55	80/85
	Likelihood ratio	8.5	1.7	5.3
**PRO-C4**	AUC	0.91***	0.81**	0.66
	Sensitivity/specificity %	80/90	75/85	50/80
	Likelihood ratio	8.0	5.0	2.5
**PRO-C5**	AUC	0.91***	79/85	0.68
	Sensitivity/specificity %	95/85	79/85	55/75
	Likelihood ratio	6.3	5.3	2.2
**PRO-C6**	AUC	0.99***	0.87***	0.88***
	Sensitivity/specificity %	95/95	79/85	70/90
	Likelihood ratio	19.0	3.8	7.0

Bonferroni correction was used to find the new critical p-value < 0.0045.

*: <0.0045

**: <0.0005

***: <0.0001.

Ctrl: asymptomatic controls

Furthermore, an AUC of 0.88 (90/70) for early diffuse vs. late diffuse SSc for PRO-C6 was found. C4M, PRO-C3, PRO-C4, and PRO-C5 were all found to have AUC’s of >0.90 when classifying asymptomatic controls vs. early diffuse SSc.

The biomarker examined in this study needs to be further validated in other cohorts. As this was a small, exploratory cohort there are limitations to this study. The cohort consisted of 20 early and 20 late diffuse SSc patients. More patients are warranted to determine if these biomarkers performs as well in a larger cohort. Furthermore, patients were only examined at one time point and it is therefore not possible to examine the biomarkers over time and in regards to progression. Lastly, treatments and organ involvement was not taken into account. These might affect the biomarkers, as the collagens are present in other tissues as well.

## Discussion

There is a lack of objective biomarkers that stratify patients, predict organ involvement, and identify progressors. Furthermore, SSc is still a disease of unknown etiology and a greater understanding of the disease progression is still need for the development of better treatments. In this study, we found that ECM biomarkers levels were increased in diffuse SSc, especially in early diffuse SSc. In addition, we found that several of these biomarkers have potential to be diagnostic tools in SSc and distinguishing between early and/or late diffuse SSc compared to asymptomatic controls. PRO-C6 was the best biomarker to differentiate between early and late diffuse SSc with an AUC of 0.88 (sensitivity/specificity of 70%/90%). Turnover of type VI collagen (PRO-C6 and C6M, respectively) were the best biomarkers to differentiate between asymptomatic and early diffuse SSc with AUCs of 0.99 (95%/95%) and 0.91 (90%/90%), respectively.

A biomarker of the N-terminal pro-peptide of type III collagen (PIIINP) was shown to be up-regulated in SSc patients compared to healthy patients and proposed as a diagnostic and treatment efficacy biomarker [19, 20, 22, 32]. However, the PIIINP assay detects both degradation and formation. The pro-peptide is not always cleaved of, thereby attached to the molecule resulting in thin fibrils with abnormally cross-links and prone to rapid metabolic turnover[33, 34]. This warrants investigation of PRO-C3 in SSc as it is a true formation biomarker detecting the amino acid sequence exposed, when the pro-peptide is cleaved of. In the current study, we found that PRO-C3 was increased in SSc compared to asymptomatic controls, but only in the early diffuse SSc patients. PRO-C3 was furthermore the best diagnostic biomarker between early and late diffuse SSc. With these results, PRO-C3 appears to be a better biomarker of type III collagen formation in SSc.

Type VI collagen is suggested to play a large role in the assembly of the collagenous matrix in the dermis[35]. Our study underlines the importance of type VI collagen in SSc as biomarkers of formation and degradation of type VI collagen was the most efficient biomarkers to differentiate the three groups assessed. In addition, the diagnostic assessment of PRO-C6 gave an AUC of 0.99 (sensitivity/specificity of 95%/95%), when assessing early diffuse SSc and asymptomatic controls. This correlates with the increased expression of type VI collagen genes have been observed in SSc patients[36]. Further analysis is needed to confirm these results, but biomarkers of type VI collagen appears as promising diagnostic biomarkers for SSc.

## Conclusion

In this study, protein fingerprint formation biomarkers of ECM turnover were shown to be significantly different between asymptomatic controls and SSc (early, late or early+late). Type VI collagen biomarkers (C6M and PRO-C6) showed to have high diagnostic power together with several other biomarkers. This study indicates that serological assessment of ECM turnover is of great interest in SSc, both for investigation of disease pathogenesis, but also as diagnostic tools.

## Supporting information

S1 Table Biomarker levels in samples. The biomarker levels in samples and their diagnosis. Shown in ng/ml.
(XLSX)
